# Genetic analysis of cilia and centrosome function in the development of the mouse embryo

**DOI:** 10.1186/2046-2530-1-S1-O19

**Published:** 2012-11-16

**Authors:** KV Anderson, B Bazzi

**Affiliations:** 1 Sloan-Kettering Institute, New York, USA

## 

Work in the past decade has revealed that cilia and centrosomes, specialized microtubule-based organelles, can act as centers for developmental signaling pathways. It is now well-established that Sonic hedgehog (Shh) signaling depends on the primary cilium. Cilia are templated by the basal body, a specialized centrosomes, and it has been suggested that centrosomes may act as signaling centers important in the Wnt and other signaling pathways. To test the role of centrosomes in mouse development, we are analyzing the phenotypes of embryos that lack the *Sas4* gene (also called *Cenpj* or *Cpap*), which is essential for centriole duplication. *Sas4* mutant embryos lack cilia and centrosomes, but survive to e9.0. Shh signaling is blocked in *Sas4* mutants, as expected because of the absence of cilia, but we have not detected defects in Wnt signaling in the mutants. *Sas4* mutants die at an early stage of development than embryos that lack cilia, indicating that the centriole has functions in addition to templating cilia. *Sas4* mutants show a high rate of apoptosis, and the early lethality of *Sas4* embryos is rescued by removal of p53. We are currently investigating the p53-dependent pathways activated in *Sas4* mutants that lead to cell death. Because human genetic diseases that disrupt the centrosome cause microcephaly, we are comparing the roles of cilia and centrosomes in patterning and cell behavior in the developing brain. These experiments will define the roles of cilia and centrosomes in the control asymmetric cell division and migration in the cortex.

